# Simvastatin Modulates Mesenchymal Stromal Cell Proliferation and Gene Expression

**DOI:** 10.1371/journal.pone.0120137

**Published:** 2015-04-13

**Authors:** Dalila Lucíola Zanette, Julio Cesar Cetrulo Lorenzi, Rodrigo Alexandre Panepucci, Patricia Vianna Bonini Palma, Daiane Fernanda dos Santos, Karen Lima Prata, Wilson Araújo Silva

**Affiliations:** 1 Department of Genetics, Ribeirão Preto Medical School, University of São Paulo, Ribeirão Preto, São Paulo, Brazil; 2 Regional Blood Center of Ribeirão Preto and Center for Cell-Based Therapy-CEPID/FAPESP, Ribeirão Preto, São Paulo, Brazil; 3 National Institute of Science and Technology in Stem cell and Cell Therapy, Ribeirão Preto, Brazil; Center for Molecular Biotechnology, ITALY

## Abstract

Statins are widely used hypocholesterolemic drugs that block the mevalonate pathway, responsible for the biosysnthesis of cholesterol. However, statins also have pleiotropic effects that interfere with several signaling pathways. Mesenchymal stromal cells (MSC) are a heterogeneous mixture of cells that can be isolated from a variety of tissues and are identified by the expression of a panel of surface markers and by their ability to differentiate *in vitro* into osteocytes, adipocytes and chondrocytes. MSC were isolated from amniotic membranes and bone marrows and characterized based on ISCT (International Society for Cell Therapy) minimal criteria. Simvastatin-treated cells and controls were directly assayed by CFSE (Carboxyfluorescein diacetate succinimidyl ester) staining to assess their cell proliferation and their RNA was used for microarray analyses and quantitative PCR (qPCR). These MSC were also evaluated for their ability to inhibit PBMC (peripheral blood mononuclear cells) proliferation. We show here that simvastatin negatively modulates MSC proliferation in a dose-dependent way and regulates the expression of proliferation-related genes. Importantly, we observed that simvastatin increased the percentage of a subset of smaller MSC, which also were actively proliferating. The association of MSC decreased size with increased pluripotency and the accumulating evidence that statins may prevent cellular senescence led us to hypothesize that simvastatin induces a smaller subpopulation that may have increased ability to maintain the entire pool of MSC and also to protect them from cellular senescence induced by long-term cultures/passages *in vitro*. These results may be important to better understand the pleiotropic effects of statins and its effects on the biology of cells with regenerative potential.

## Introduction

Statins are widely used hypocholesterolemic drugs that block the activity of 3-hydroxy-3-methyl-glutaryl-coenzyme A (HMG-CoA) reductase, an enzyme that catalyzes the production of mevalonate, the first step in cholesterol biosynthetic pathway [1]. Intermediates upstream cholesterol also are affected, mainly 15-carbon farnesylpyrophosphate (FPP) and 20-carbon geranylgeranyl pyrophosphate (GGPP). These are called isoprenoids and function as adjuncts in prenylation, a post-translational modification that consists in the addition of a lipophilic phenyl group to the proteins. This group enables their anchorage to the cell membrane, which is essential for the activity of several cell-signaling proteins. Prenylation occurs mainly on proteins containing a carboxy-terminal CaaX motif (C is a cysteine, *a* is an aliphatic aminoacid and X is any aminoacid). Examples of prenylated proteins include about 40 members of small GTPase famlily of molecular switch proteins, such as cell division cycle 42 (CDC42), RAC, RAS homologue (RHO) and RAB family of RAS-related G-proteins, although the these latter do not have a CaaX motif. Given the central role of all these proteins, statins are known to interfere with several signaling pathways, especially in the immune response [2].

Mesenchymal stromal cells (MSC) are isolated from a variety of tissues and under culture they are spindle-shaped adherent cells that can differentiate *in vitro* into osteocytes, adipocytes and condrocytes. These observations suggested that MSC were responsible for the normal turnover and maintenance of mesenchymal tissues and tissue regeneration after injury [3]. Usually MSC are so called when the cultured cells fulfill the minimal criteria of BMSC defined by International Society for Cell Therapy (ISCT), based on their surface markers and differentiation potential [4]. Despite this, MSC preparations are a heterogeneous mixture of different cell subpopulations in many aspects, as overviewed by Schellenberg and collaborators [5] and discussed in [6]. MSC are able to inhibit peripheral blood mononuclear cell and lymphocyte proliferation *in vitro* [7,8]. MSC are thought to escape immune recognition by alloreactive cells or at least they exhibit low immunogenicity. These properties are extremely important for MSC therapeutic use in allogeneic transplantation [9]. MSC are currently used in bone marrow transplantation to improve engraftment and to prevent graft-versus-host disease (GVHD) [8].

Statins are one of the most commonly used drugs in the world to decrease cholesterol levels but its immunomodulatory properties led us to investigate the effects of simvastatin on MSC, given the impact that those effects may have to the use of MSC in stem cell therapy and in the prevention of GVHD in hematopoietic stem cell transplantation.

In this report, we show that simvastatin negatively modulates MSC proliferation in a dose-dependent way, as directly seen by proliferation assays and reinforced by the modulation of proliferation-related genes observed in microarray results. Also, simvastatin seems to affect not only MSC proliferation, but also their size, in a way that the smaller MSC show increased proliferation activity. This could be interpreted in at least two ways: simvastatin may induce the proliferation of a smaller MSC subset; or decrease MSC size. Despite this, the overall diminished proliferation did not affect the ability of MSC to inhibit PBMC (peripheral blood monocytic cells) proliferation. Given the wide use of statins, the effects of these drugs on MSC can be of extreme importance in the context of MSC transplantation, in GVHD prevention and also in the homeostasis of mesenchymal tissues.

## Materials and Methods

### Ethics Statement

All samples were obtained after informed consent had been obtained from the patients and the study was approved by the institutional ethics committee by the number 12855–08.

#### Isolation of Mesenchymal Stromal Cells (MSC) from human amniotic membranes

Mesenchymal Stromal Cells (MSC) were isolated from term human placenta amniotic membranes (AM) and from bone marrow. Amniotic membranes were obtained after cesarean sections of healthy pregnancies and bone marrows from leftovers of bone marrow donors.

Bone marrow MSC (BM-MSC) were isolated as previously described [10]. Placentas were collected right after cesarean sections and amniotic membrane was carefully peeled from the underlying chorion and digested with Collagenase I (Sigma St. Louis, MO, USA) (1 mg/mL) and filtered through a 100μm strainer (BD Falcon, San Jose, CA, USA). The cell suspension obtained was counted and plated at an average density of 1x10^5^/cm^2^ in *Minimum Essential Medium* (*MEM*) *Alpha* Medium (Invitrogen, Carlsbad, CA, USA) supplemented with 10% FBS, at 37°C with 5% CO_2_ until semi-confluence was reached. At this point, cells were detached and further considered as the first passage. Cells were then cultured for several passages, always in low densities (2,000 cells/cm^2^) and in the presence of the fetal bovine serum from the same brand and lot (Hyclone lot number ATG32533). All the isolated MSCs were fusiform, plastic adherent cells and expressed variable levels and fulfilled the ISCT minimal criteria [4] regarding surface markers and osteogenic and adipogenic differentiation (S1 Fig). Except for microarray analysis, all other experiments were performed with both bone marrow and amniotic membrane MSC to address if there were differences between those cells in response to simvastatin.

### *In vitro* cell cultures

Nine to eleven different AM-MSC samples and one BM-MSC were used for *in vitro* treatments with sodium simvastatin salt (Calbiochem, La Jolla, CA, USA/Cat.No 567021) for 72 hours at drug concentrations ranging from 1 to 5μM. Samples were also treated with a combination of 5μM simvastatin plus 5μM GGPP or simvastatin plus 100μM activated L-mevalonate (all from Sigma St. Louis, MO, USA). Control cells were exposed only to the drug vehicle (absolute ethanol, maximum final volume 0.5μL per 1 mL of culture). Cells were treated between 6^th^ and 8^th^ passages, and treatments started when cells were 40% confluent, in average. After 72 hour-treatments, cells were harvested and collected for flow cytometry analyses and RNA isolation.

### Flow cytometry analysis for MSC characterization

AM-MSC and BM-MSC surface markers characterization was performed by flow cytometry using monoclonal antibodies against CD105, CD45, CD73, CD34, CD90, CD14, CD19, HLA-I and HLA-DR (Becton-Dickinson, San Jose, CA, USA). All analyses were performed in a FACScalibur equipment using the Cell Quest software (both from Becton-Dickinson, San Jose, CA, USA). The equipment was calibrated for the acquisition of 10,000 events per labeling. For each sample, Simultest (Becton-Dickinson, San Jose, CA, USA) was used as a control isotype to eliminate nonspecific labeling. Cell viability was assessed with Annexin V-FITC and Propidium Iodide (PI) kit (Becton-Dickinson, San Jose, CA, USA).

### *In vitro* suppression of PBMC proliferation by MSC

We tested whether MSC were able to suppress activated PBMC proliferation *in vitro* and also if simvastatin could affect this ability. For this purpose, we co-cultured simvastatin pre-treated and control MSC with phytohemaglutinin (PHA)-activated PBMC. Briefly, semi-confluent MSCs were treated with 0.1, 1 and 5μM of simvastatin for 72 hours in 24-well plates. At this point, MSC were extensively washed to completely remove simvastatin from these cultures. PBMC were isolated using a Ficoll-Paque density centrifugation method and were activated with 10μg/mL of PHA (Sigma-Aldrich). These activated PBMC were stained with 2.5μL of a 10mM stock solution of CFDA-SE (Molecular Probes), also known as CFSE (carboxyfluorescein diacetate succinimidyl ester) for 5 minutes at 37°C, followed by 3 washes to remove the excess of CFSE. After that, an equal volume of FBS was added and cells were incubated for 1 minute at room temperature. Cells were pelleted and washed twice with the regular culture media to remove the excess of CFSE. PBMC were then added to the MSC cultures in a proportion of 5 PBMC to 1 MSC and cultured for 5 days at 37°C with 5% CO_2_. CFSE is a cell-permeant fluorescein-based dye CFSE covalently attaches to cytoplasmic components of cells, resulting in uniform bright fluorescence. Upon cell division, the dye is distributed equally between daughter cells, allowing the resolution of up to eight cycles of cell division by flow cytometry [11]. One day after staining, the initial CFSE fluorescence intensity (maximum) was evaluated, by using an aliquot of stained cells. This measure of fluorescence is the maximum of CFSE intensity. Also on day 1, we determined the background intensity by measuring the CFSE intensity for non-stained cells. The same acquisition parameters were used to access the CFSE intensity after 72 hours (day 5). The proliferating gate was fixed between the maximum CFSE intensity value, measured on day 1 and the background CFSE intensity, also measured on day 1, but for non-stained cells. The fluorescence measured in this proliferating gate corresponds to the proliferative activity of PBMC during the 5-day experiment. After the acquisition, all gating and data analyzes were performed using the FlowJo 8.7 software.

### CFDA staining and analysis of MSC proliferation by flow cytometry

**Fig 1:**
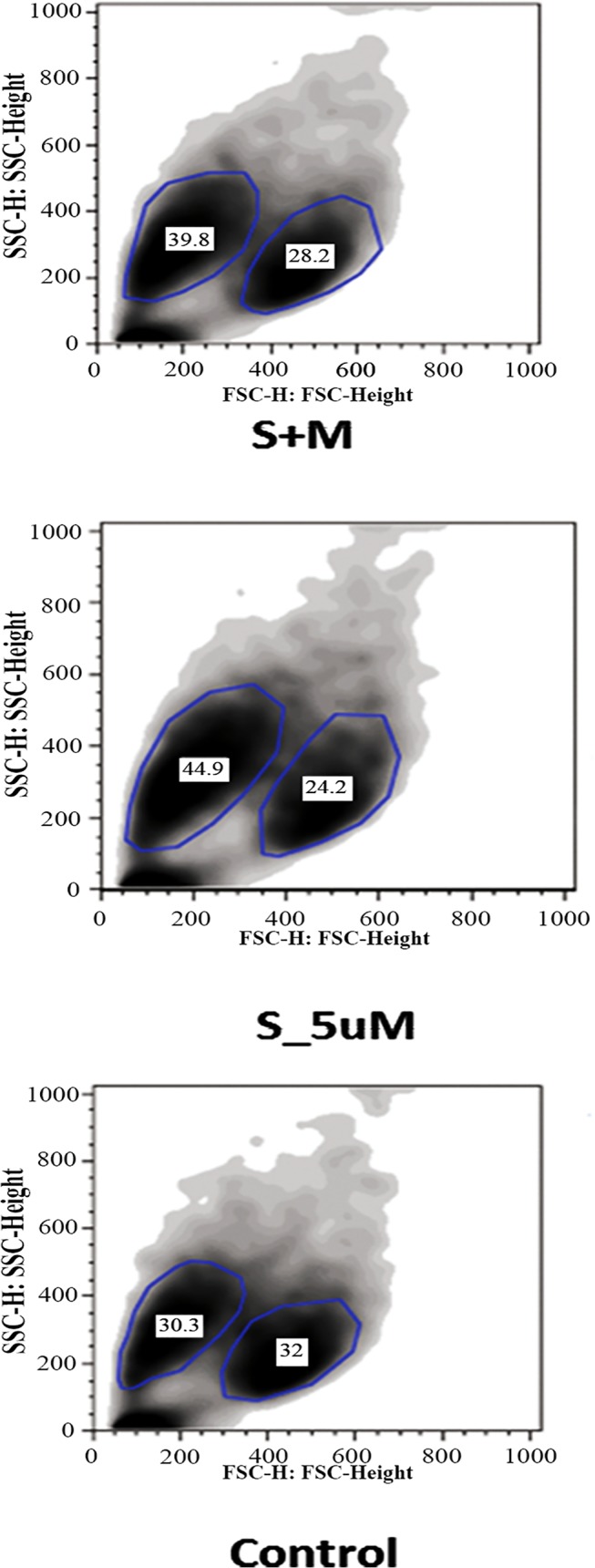
MSC subpopulations by size (FSC) and complexity (SSC). The different subsets are surrounded by a blue line drawn around them and the values correspond to the percentage of each in this example. S_5uM, MSC treated with simvastatin in the concentration of 5μM. S+M, MSC treated with 5μM of simvastatin and 100μM of activated L-Mevalonate (M).

Based on the previous report, from Urbani and collaborators [12], we applied a similar CFSE staining protocol was used for MSC, to evaluate the effects of simvastatin on their proliferation. CFSE-stained MSC were seeded on 24-well plates, at a density of 2x10^4^ cells/mL and further cultured at 37°C with 5% CO_2_ for 5 days. Twelve hours after plating, cells were subjected to different simvastatin concentrations and also to mevalonate or GGPP. The general gating strategy for proliferating cells was the same used for PBMC, but in the case of proliferating MSC, different subpopulations were analyzed based on size and complexity, as shown in Fig 1B, exemplified in S2 Fig and further discussed in the results section. After the acquisition, all gates and data analyzes were performed using the FlowJo 8.7 software.

### RNA isolation

Cells were detached with Tryple Express (Invitrogen, Carlsbad, CA, USA), washed twice with PBS and their RNA was isolated using TriZOL LS Reagent, according to manufacturer’s instructions. The RNAs used for microarrays were checked for quality in the Bioanalyzer equipment (Agilent Technologies). Only RNAs with RIN (RNA Integrity Number) values greater than 7 were used for microarrays.

### Microarrays

Total RNA was used for microarrays of four different AM-MSC samples were used, 4 untreated, control cells and their 1μM simvastatin-treated counterparts totalizing 8 microarrays. The microarray platform was the Whole Human Genome Oligo Microarray Kit (G4112F, Agilent Technologies, Palo Alto, CA, USA). Microarray normalization and quality control was performed using the R statistical environment (http://www.r-project.org) with the Agi4x44PreProcess package downloaded from the Bioconductor web site (http://bioconductor.org/). Data were modified to be compatible for the Agi4x44PreProcess packages. The normalization and filtering steps were based on those described in the Agi4x44 PreProcess instructions.

### Validation of microarray data

Seven transcripts were selected from the microarray analysis for validation by quantitative PCR (qPCR) using TaqMan primers and probes (all purchased from Applied Biosystems, Assay IDs listed in S3 Table). Briefly, cDNA was obtained with High Capacity cDNA Archive kit (PN 4368813, Applied Biosystems) following the manufacturer’s instructions. All reactions were performed in duplicates and run in a 7500 Sequence Detection System (Applied Biosystems), with standard TaqMan conditions (1 cycle of 10 min at 95°C, followed by 40 cycles of 94°C, 15 s; 60°C, 1 min) and analyzed with the ABI-7500 SDS software package. Total RNA input was normalized based on the geometric mean of Ct values for two endogenous controls, TBP and HPRT genes (4326322E and 4326321E, respectively, Applied Biosystems). The fold change was calculated using 2^-ΔCt^ method [13].

### Pathway analysis

MetaCore version 6.16 (Thomson Reuters) was used to analyze the signaling pathways modulated by simvastatin. The input genes were the top 300 down- and up-regulated genes found in the microarray results. Among several tools offered by this software, we used mainly the following: Enrichment Analysis, Pathway Maps and Key Transcription Factors and Target Genes.

### Statistical analyses

Validation of microarrays by qPCR and CFSE cell proliferation assays were analyzed using non-parametric One-way Anova, Friedman test followed by Dunns post-test.

## Results

### Effects of simvastatin on gene expression

**Fig 2:**
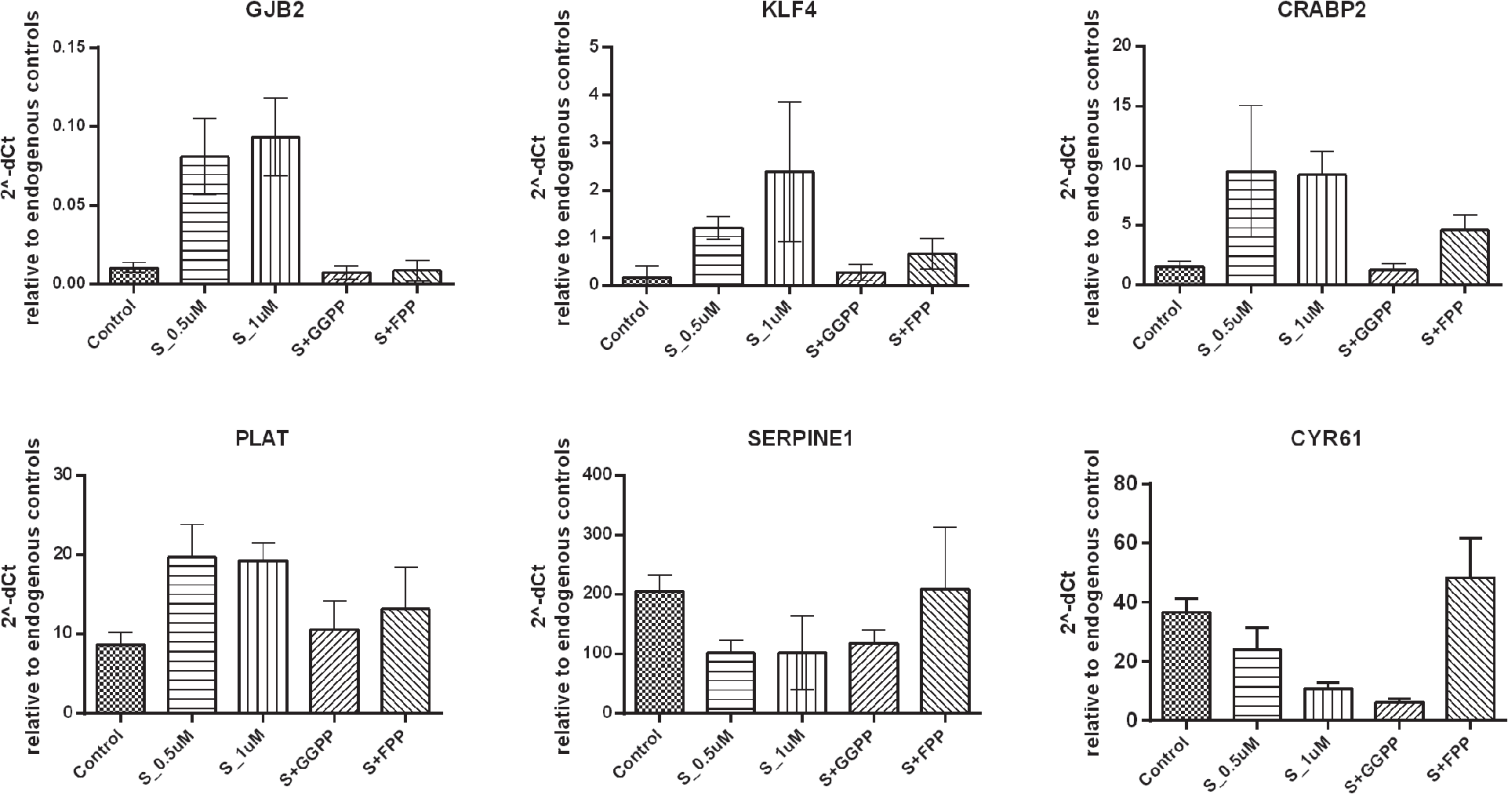
Validation of microarray experiments by quantitative real time PCR (qRT-PCR) for selected genes. The graph shows the median of 2^-dCt values, agains the geometric mean of the dCt of two reference genes. Control refers to MSC exposed only to the vehicle (absolute ethanol). S_1Um and S_5uM: MSC treated with simvastatin in the concentration of 1μM and 5μM, respectively. S+FPP and S+GGPP, MSC treated with 5μM of simvastatin and 5μM of FPP (15-carbon farnesylpyrophosphate) and 5μM of GGPP (20-carbon geranylgeranyl pyrophosphate), respectively. S+M, MSC treated with 5μM of simvastatin and 100μM of activated L-Mevalonate (M).

The top 25 up- and down-regulated genes, according to the microarray results, are shown in S1 and S2 Tables, respectively. Six genes were chosen for validation by qRT-PCR (GJB2: gap junction protein, beta 2, 26kDa; KLF4: Kruppel-like factor 4; CRABP2: cellular retinoic acid binding protein 2; PLAT: plasminogen activator, tissue; SERPINE1: serpin peptidase inhibitor, clade E and CYR61: cysteine-rich, angiogenic inducer, 61), and showed the same expression pattern seen in the microarrays (Fig 2). Furthermore, to confirm the specificity of simvastatin effect on gene expression, we added two intermediates of the mevalonate pathway, GGPP and FPP. The addition of GGPP completely abolished the effects of simvastatin on KLF4, CRABP2, PLAT and SERPINE1 gene expression, while FPP showed only a partial reversion on these genes. The exception was CYR61, for which the effects of simvastatin were reversed only by the addition of FPP. AM-MSC were used for microarrays, but BM-MSC and AM-MSC were used for qPCR validation and both confirmed microarray findings.

**Fig 3:**
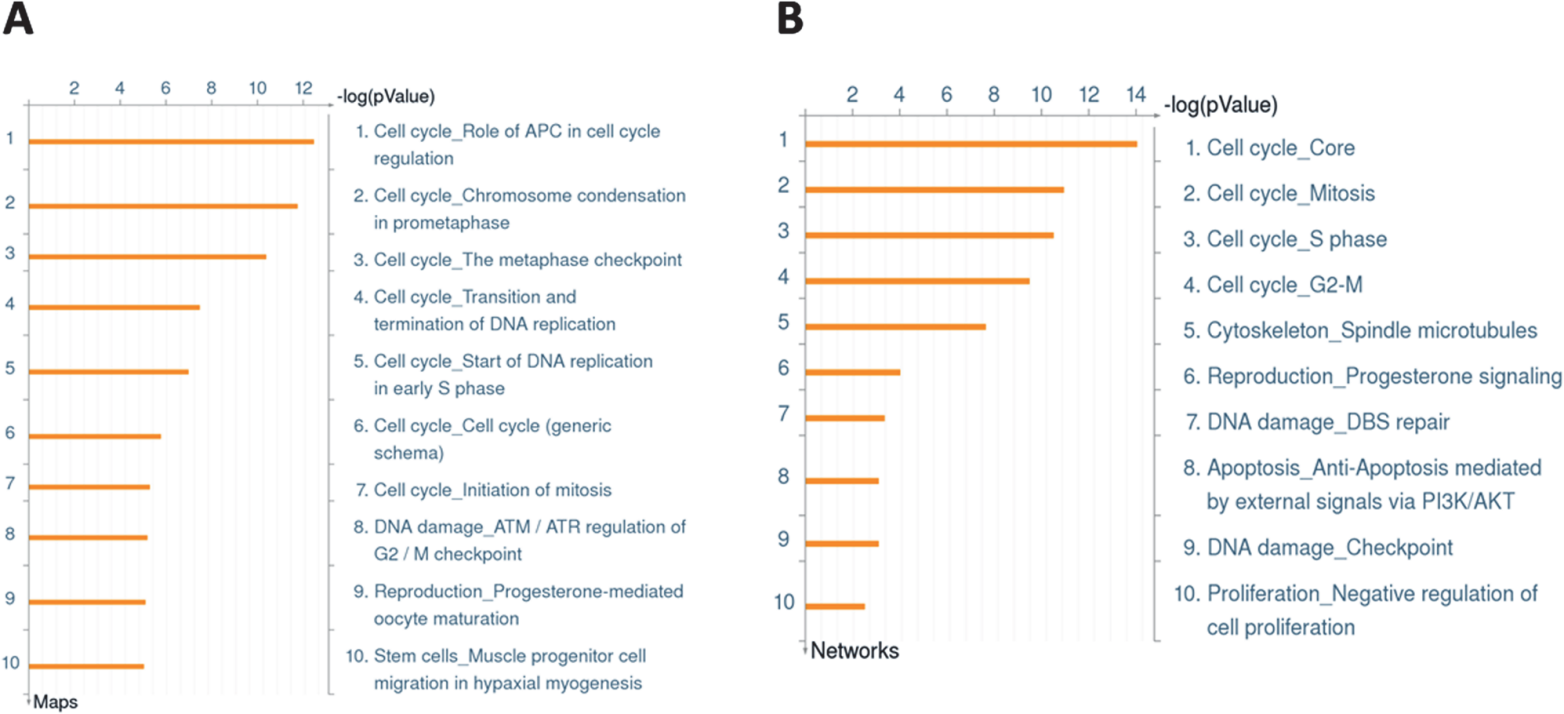
MetaCore analyses of the the top 300 up and down-regulated genes. A. Enrichment Analysis for the Top 10 Pathways. B. Enrichment Analysis for the Top 10 Process Networks.

The top 300 up and down-modulated genes were analyzed with MetaCore version 6.16 to predict the signaling pathways affected by simvastatin. This software offers many different tools to understand which pathways are affected by the differences in gene expression found in the microarray data. We were initially interested in gene expression modifications induced by simvastatin, aiming to find out which cell properties could be modified by this drug. By using any of MetaCore tools, the results repeatedly showed that simvastatin affects pathways or networks related to cell cycle, cell proliferation and DNA replication/repair. Seven out of the ten 10 top regulated Pathways are related to cell cycle (Fig 3A). Four out of ten of the top regulated Processes were also related to cell cycle, two were associated with DNA damage and one with negative regulation of cell proliferation (Fig 3B). Most of the genes found within those pathways and processes were down-regulated by simvastatin, such as CDC25A, CDC20, CCNE2, CNNA, CDK1 and PLK1, as exemplified by the top pathway, Role of APC in cell cycle regulation. Despite the indication of apoptosis regulation, as already mentioned, we could not find any differences in cell viability in treated versus control cells, as assessed by Annexin V and Propidium Iodide analyses (data not shown). As these findings indicate an arrest in cell cycle that may lead to an inhibition of MSC proliferation by simvastatin, we decided to further evaluate the effects of simvastatin on MSC proliferation pattern by CFSE assays as described below.

### The ability of MSC to suppress to PBMC proliferation in vitro is not affected by simvastatin

**Fig 4:**
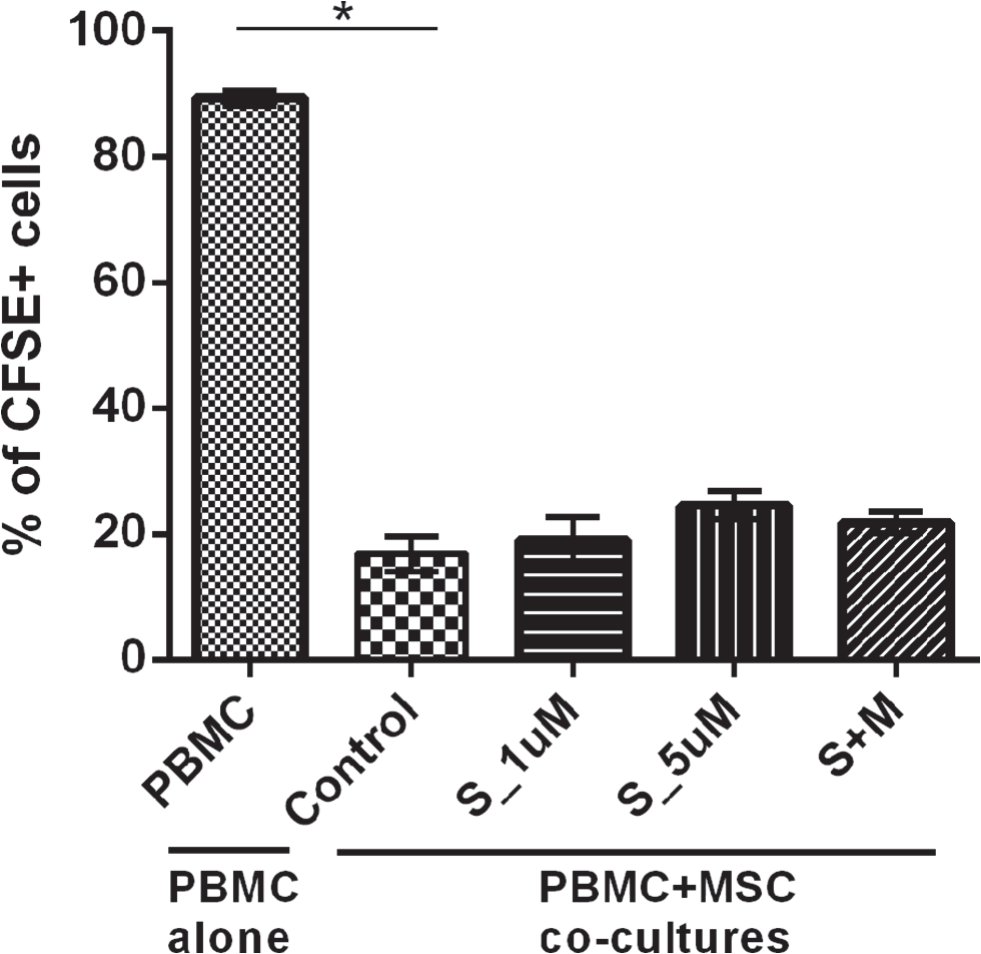
Median values of CFSE+ PBMC to assess the ability of simvastatin-treated MSC to inhibit PBMC proliferation. PBMC alone correspond to the proliferation rate of PBMC alone. Control refers to co-cultures of PBMC with MSC samples exposed only to the vehicle (absolute ethanol). S_1uM: MSC treated with simvastatin in the concentration of 1μM. S_5uM, MSC treated with simvastatin in the concentration of 5μM. S+M, MSC treated with 5μM of simvastatin and 100μM of activated L-Mevalonate (M).

Both AM-MSC and BM-MSC significantly inhibited PBMC proliferation but no significant difference was found in the percentage of proliferating PBMC *in vitro* when simvastatin was added to the co-cultures (Fig 4), even in the highest simvastatin dose used (5μM).

### Simvastatin modulates MSC proliferation

**Fig 5:**
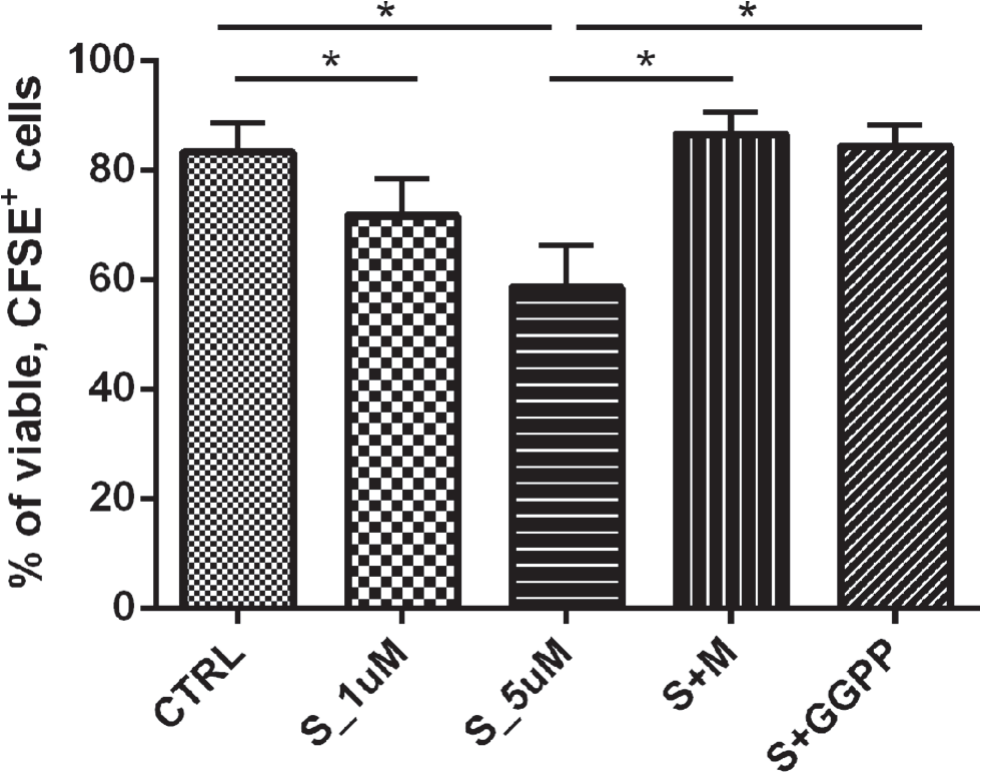
CFSE proliferation assays of MSC. The values correspond to the median percentage of viable, CFSE+ cells. S_1uM: MSC treated with simvastatin in the concentration of 1μM. S_5uM, MSC treated with simvastatin in the concentration of 5μM. S+M, MSC treated with 5μM of simvastatin and 100μM of activated L-Mevalonate (M). S+GGPP, MSC treated with 5μM of simvastatin and 5μM of GGPP (20-carbon geranylgeranyl pyrophosphate).

Simvastatin reduced AM-MSC and BM-MSC proliferation in a dose-dependent way, as observed by the percentage of proliferating cells in the CFSE assays and did not affect cell viability even with the higher dose used (Annexin/Propidium Iodide staining, data not shown). A smaller dose (1μM) of simvastatin significantly reduced cell proliferation (p<0.05), although the effect was more pronounced with 5 μM, indicating a dose-dependent fashion. Importantly, these effects were totally reversed by the addition of L-mevalonate and partially reversed by GGPP (Fig 5).

### Simvastatin effect on MSC size and complexity

**Fig 6:**
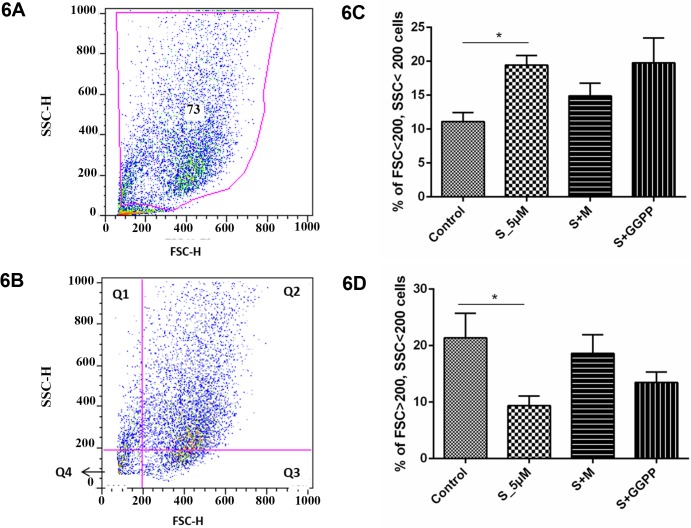
Analysis of MSC proliferation for the quadrant of small (FSC^lo^) and low complexity (SSC^lo^) cells. S_1uM: MSC treated with simvastatin in the concentration of 1μM. S_5uM, MSC treated with simvastatin in the concentration of 5μM. S+M, MSC treated with 5μM of simvastatin and 100μM of activated L-Mevalonate (M). S+GGPP, MSC treated with 5μM of simvastatin and 5μM of GGPP (20-carbon geranylgeranyl pyrophosphate). A) gating strategy to remove debril and dead cells; B) FSC and SSC quadrants definition; C) Percentages of cells with FSC and SSC< 200; D) Percentages of cells with FSC and SSC> 200.

During proliferation analysis by flow cytometry, different subpopulations wereidentified by size and complexity differences and most interestingly, simvastatin seemed to increase the percentage of smaller cells and this effect was reversed by the addition of Mevalonate, as can be seen in Fig 1. This finding led us to further analyze different cells subsets. To separate cell subpopulations more precisely, a gate was built excluding possible dead cells and debris. Then a quadrant was fixed in FSC 200 and SSC 200, dividing the scatter gram in four subpopulations according to cell size and complexity: Q1 (small, high complexity cells), Q2 (large, high complexity cells), Q3 (large, low complexity cells) and Q4 (small, low complexity cells). The cells within the high complexity subset maintained their size, which did not vary between treated and control MSC (data not shown). However, within the low complexity subset, simvastatin-treated cells showed a higher percentage of small cells at the expense of the large cell subset (p<0.05). Furthermore, this effect was almost completely abrogated by the addition of mevalonate, and partially reversed by GGPP addition (Fig 6).

### Simvastatin effects depending on cell size

**Fig 7:**
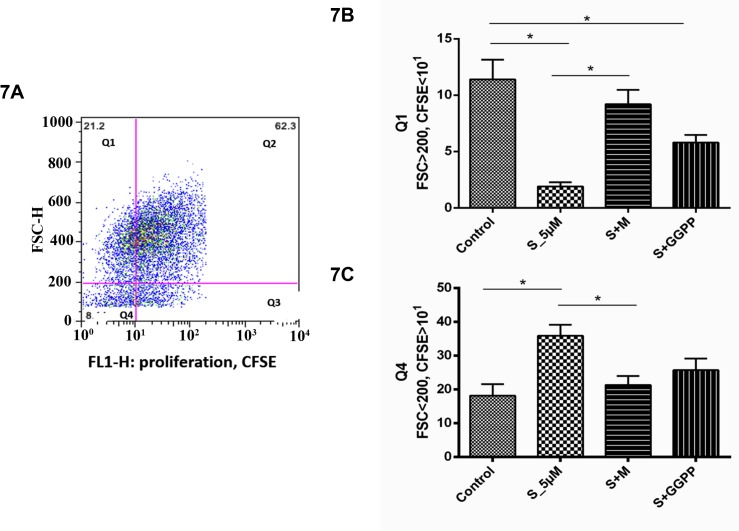
Analysis of MSC proliferation for A) the quadrant of small (FSC^lo^) and proliferative (CFSE^hi^) MSC and for B) quadrant of large (FSC^hi^) and non-proliferative (CFSE^lo^) MSC. S_1uM: MSC treated with simvastatin in the concentration of 1μM. S_5uM, MSC treated with simvastatin in the concentration of 5μM. S+M, MSC treated with 5μM of simvastatin and 100μM of activated L-Mevalonate (M). S+GGPP, MSC treated with 5μM of simvastatin and 5μM of GGPP (20-carbon geranylgeranyl pyrophosphate).

The proliferating subset of cells was further analyzed regarding cell size and complexity by creating a quadrant, fixed on FSC 200 and CFSE intensity 1, obtaining 4 different cell subsets: Q1 (large, non-proliferating cells), Q2 (large, proliferating cells), Q3 (small, proliferating cells) and Q4 (small, non-proliferating cells). Simvastatin increased the percentage of small cells, showing CFSE intensity >1 (Fig 7A), whilst decreased the percentage of large, CFSE low cells (Fig 7B) (p<0.05). This effect was reversed by the addition of mevalonate and partially reversed by GGPP. The other cell subsets were not significantly affected (data not shown).

## Discussion

In this report, we show that simvastatin affect MSC proliferation and interferes in the expression of genes involved with cell proliferation and cell cycle progression. We confirmed these findings by directly assessing cell proliferation by CFSE assays. Importantly, the effects of simvastatin on both gene expression and proliferation assays were reversed by mevalonate and partly reversed by GGPP, implying that the effects were a direct consequence of the inhibition of mevalonate pathway by the drug and, at least in part, were mediated by geranylgeranylpirophosfate isoprenoids.

The effects of simvastatin on cell proliferation have been well documented in a variety of cancer, leukemia and immune diseases, in which statins were mostly shown to inhibit cell proliferation [14,15,16,17,18,19], although there is a conflicting report [15]. Furthermore, some studies claim that simvastatin exclusively affects cancer cell proliferation [20,21]. Besides this, there is only scarce information about its effects on normal cells, in which the effects of statins on cell proliferation were usually analyzed after the induction of cells with combinations of drugs and stimulating molecules [22,23]. Regarding MSC, there is only one report showing that high doses of another statin, fluvastatin (10μM), induced morphological changes, abrogation of the S-phase of cell cycle and detachment from the substrate that ultimately led to BM-MSC death [24]. It was also reported that 10 μM simvastatin decreased human endometrial stromal cell proliferation, but this high dose also induced apoptosis in that study [25]. Here we report that 5 μM of simvastatin decreases MSC proliferation without any effects on their ability to inhibit PBMC proliferation or induction of apoptosis. The down-modulation of several genes involved in cell cycle progression and cell proliferation, such as PLK1, CDC25A, CDC20, CDC6, Cyclins A1 and E2 and CDK1 is noteworthy and may help to explain the effects of simvastatin found on cell proliferation assays. The induction of cell cycle arrest by statins has already been documented but there is some controversy if S, G1 or G2/M phase are affected, as briefly discussed in [26]. The majority of the reports on statin induced cell cycle arrest and inhibition of cell proliferation were performed in cancer cell lines and show that these effects are usually accompanied by induction of apoptosis [27]. Here, MSC viability was not affected by simvastatin, even at the higher dose used. This may be explained by the different effect of statins on the apoptosis of cancer versus normal cells, as reported by Spampanato [19] and by Shy and collaborators [28], showing that statin induces apoptosis in cancer cells, but does not affect normal cells in the same conditions.

We also found differential effects of simvastatin on the proliferation of MSC depending on their size (FSC) and complexity (SSC) that require special attention. It has long been known that *in vitro* cultured MSC comprise a heterogeneous cell population. Colter and colleagues showed that MSC plated in low-densities contain a subpopulation of small and rapidly self-renewing cells, associating MSC decreased size with increased pluripotency [29]. Another study showed that smaller MSC had increased expression of mesenchymal markers, higher proliferative activity and lower expression of a senescence-associated marker [30]. Whitfield and colleagues confirmed previous observations that MSC are smaller at low passages than in higher passages and showed that actively dividing MSC maintain their size, while those that stop proliferating grow larger over passages [31]. We show here that simvastatin increases the percentage of smaller cells within MSC total population. The percentage of smaller cells also showing higher CFSE intensity was also increased by simvastatin, possibly indicating a selective induction of the proliferative activity of a subset of cells showing smaller size, while it inhibits the proliferation of the larger cells. This skewing could be also be caused by a reduction in cells size, induced by the drug. The first explanation can be associated to the modulation of cells cycle and cell proliferation genes, as we found in the microarray analyses. The second argument could be explained by the induction of morphological changes by statins [24]. Hence, it is reasonable to suggest that simvastatin alters MSC proliferation and increases the percentage of smaller and actively dividing cells, whilst maintains the absolute pool of cells. In this way, simvastatin reduces total MSC proliferation activity, but does not affect the absolute number of cells, which may explain why simvastatin did not affect MSC ability to inhibit PBMC proliferation, despite the overall inhibition of MSC proliferation.

CRABP2, KLF4, PLAT, SERPINE and CYR61 genes were found to be highly modulated by simvastatin treatment and were chosen to be validated by qPCR. Importantly, the reversibility of simvastatin effects by mevalonate and/or FPP/GGPP was demonstrated here for these genes. The induction of CRABP2 by simvastatin was recently shown and was accompanied by the reduced cell proliferation in human endometrial stromal cells. In the same study the authors found that simvastatin interacts with retinoic acid (RA) signaling pathways, demonstrating a potentiation of RA effects. As CRABP2 shuttles RA from cytosol to the nucleus and directs RA toward its receptor RAR, it may be in the interface of the synergy between statins and RA [25]. This synergy has also been demonstrated in acute promyelocytic leukemia [32]. Consequently, it is reasonable to think that statins could make MSC more sensitive to RA, which may have implications in the facilitation of MSC neuronal differentiation observed upon all-trans retinoic acid pre-induction [33]. It was also demonstrated that CRABP2 is downregulated MSC in higher passages as compared with early passages, which may indicate its regulation upon cell senescence [34].

KLF4 is a zinc finger transcription factor involved in several important cellular processes, and is known to inhibit cell proliferation, to regulate cell cycle and to be important for the maintenance of genetic stability [35] [36]. KLF4 has been described as a regulator of MSC fate, showing higher expression in MSC compared with differentiated fibroblasts and a decreased expression upon induction of MSC differentiation. In the same report, the authors describe a large occurrence of KLF4-binding sequences within the promoter regions of target genes highly expressed in MSC, implying a potential role of KLF4 in controlling the MSC transcriptional program [37]. These findings suggest that KLF4 is involved in the maintenance of MSC in an undifferentiated state, which makes perfect sense since KLF4 is one of the four transcription factors required to reprogram fibroblasts into induced pluripotent cells (iPSC) [38]. Hence, the induction of KLF4 gene expression found here may be one of the factors involved in the differential modulation of MSC subsets proliferation by simvastatin.

SERPINE1, also known as PAI-1 was reported to be a marker of aging and senescence because of its overexpression during replicative senescence and organismal aging [39]. More recently, SERPINE1 role in stress-induced senescence was described, where it is induced by stress, inhibits PLAT and IGFBP3, resulting in cell senescence [40]. Moreover, SERPINE1 expression was reported to increase during aging in tumor-derived fibroblasts and more importantly, the results of this study suggested that the levels of SERPINE1 might be regulated by telomere length or by telomere reconstitution activity [41]. In our study, SERPINE1 is negatively regulated in simvastatin-treated MSC, while PLAT is positively regulated, which may indicate, along with the other genes found to be modulated, that simvastatin could inhibit senescence also in the context of *in vitro* cutured MSC. Simvastatin has already been show to inhibit the expression of CYR61 in rheumatoid arthritis (RA) synovial fibroblasts, which is a beneficial effect because CYR61 is involved in the pathogenesis of RA [42]. CYR61 was shown to induce senescence, decrease cell proliferation and induce cell cycle arrest genes in muscle cells, but seems to have very diverse and opposing effects in other cells, as Du and collaborators briefly reviewed in their study [43]. We found that the addition of FPP, but not GGPP could reverse the down-modulation of CYR61 in simvastatin-treated MSC, in contrast with the report of Fromigue and colleagues in osteosarcoma [44], which may be explained by the cell-type and context dependent function and regulation of CYR61.

Ultimately, there is accumulating evidence that simvastatin and other statins may prevent or downregulate cellular senescence and proliferation [45]. A recent study conducted with 230 human subjects showed that statin treatment is associated with longer leukocyte telomere length and higher telomerase activity independently of multiple covariate, especially in older subjects [46]. We report here that simvastatin differentially modulates MSC proliferation, depending on cell size and complexity. Simvastatin seems to induce the proliferation of a subset of cells showing smaller size, while it decreases the proliferation of the subset of larger cells. We also showed that simvastatin affects the expression of genes that code proteins involved in cell cycle, proliferation and senescence pathways. The association of these results with the current knowledge about MSC cell size and statin pleiotropic effects led us to hypothesize that simvastatin differential effect on MSC proliferation or a reduction in MSC size may induce subpopulations that protect MSC from senescence induced by long term cultures/passages *in vitro*. As statins are one of the most commonly used drugs in the world and has immunomodulatory properties, its effects on cells that are used in cell therapy and transplantation are potentially interesting. In this report, we show that simvastatin negatively modulates MSC proliferation in a dose-dependent way and modulates proliferation-related genes. We also showed that simvastatin increases the percentage smaller MSC, which could be related to increased capacity to maintain their stemness potential. Given the wide use of statins, these findings are helpful to increase the current knowledge about the pleiotropic effects of statins. Moreover, the effects of commonly used drugs on MSC are important in the context of MSC transplantation, in GVHD prevention and also in the homeostasis of mesenchymal tissues.

## Supporting Information

S1 Table 25 top up-regulated genes on simvastatin-treated MSC. (PDF)

S2 Table 25 top down-regulated genes on simvastatin-treated MSC. (PDF)

S3 Table Immunophenotypical characterization of MSC by flow cytometry. Average percentage of positive and negative markers for AM-MSC and BM-MSC.
(PDF)

S1 Fig Differentiation potential of MSC: A) Example of differentiation into osteoblasts; B) Example of differentiation into adipocytes. For the differentiations, cells were cultured with specific inductors of differentiation into adipocytes (A) and osteocytes (B). Adipocyte cultures were stained with Sudan II and Scarlet stains and osteocyte cultures were stained with Von Kossa and Harris hematoxilin.
(PDF)

S2 Fig Representative images of CFSE proliferation analysis. (A) Basal CFSE staining and after co-culture staining of MSC; (B) basal CFSE staining and after co-culture staining of PBMC and (C) representative analysis of MSC CFSE experiment.
(PDF)
