# Supplementary material for: Simvastatin Modulates Mesenchymal Stromal Cell Proliferation and Gene Expression
Source: PLoS One. 2015 Apr 13;10(4):e0120137. doi: 10.1371/journal.pone.0120137 (PMC4395223; doi:10.1371/journal.pone.0120137)
Supplement: S1 Table — (PDF) [file pone.0120137.s001.pdf]

Gene Symbol	Fold change S_1uM vs Control	Description
HS3ST1	10.1	heparan sulfate (glucosamine) 3-O-sulfotransferase 1
GJB2	9.1	gap junction protein, beta 2, 26kDa
KCNN4	7.8	potassium intermediate/small conductance calcium-activated channel, subfamily N, member 4
KLF4	7.1	Kruppel-like factor 4 (gut)
MALL	7.1	mal, T-cell differentiation protein-like
CRABP2	6.9	cellular retinoic acid binding protein 2
CMKLR1	5.2	chemokine-like receptor 1
GAP43	5.2	growth associated protein 43
NGEF	5.1	neuronal guanine nucleotide exchange factor
TNXB	5.0	tenascin XB
RAB27B	5.0	RAB27B, member RAS oncogene family
AKR1B10	4.8	aldo-keto reductase family 1, member B10 (aldose reductase)
HYAL1	4.6	hyaluronoglucosaminidase 1
TMEM26	4.5	transmembrane protein 26
DDIT4	4.4	DNA-damage-inducible transcript 4
PMCH	4.3	pro-melanin-concentrating hormone

PLCD4	4.3	phospholipase C, delta 4
PMCHL1	4.2	pro-melanin-concentrating hormone-like 1, pseudogene
OLFML2A	4.2	olfactomedin-like 2A
PTGER1	4.2	prostaglandin E receptor 1 (subtype EP1)
MIAT	4.2	myocardial infarction associated transcript (non-protein coding)
AKR1B10P1	4.2	aldo-keto reductase family 1, member B10 pseudogene 1
AKR1C1	4.2	aldo-keto reductase family 1, member C1
THBD	4.1	thrombomodulin
ANKDD1A	4.0	ankyrin repeat and death domain containing 1A
