# Supplementary material for: Simvastatin Modulates Mesenchymal Stromal Cell Proliferation and Gene Expression
Source: PLoS One. 2015 Apr 13;10(4):e0120137. doi: 10.1371/journal.pone.0120137 (PMC4395223; doi:10.1371/journal.pone.0120137)
Supplement: S2 Table — (PDF) [file pone.0120137.s002.pdf]

| Gene Symbol | Fold change S_1uM vs Control | Description |
|---|---|---|
| WWC1 | -5.1 | WW and C2 domain containing 1 |
| ATAD5 | -4.5 | ATPase family, AAA domain containing 5 |
| VRTN | -4.4 | vertebrae development associated |
| KALRN | -4.4 | kalirin, RhoGEF kinase |
| CCNE2 | -4.2 | cyclin E2 |
| NEB | -4.2 | nebulin |
| C12orf54 | -4.1 | chromosome 12 open reading frame 54 |
| MCM10 | -4.1 | minichromosome maintenance complex component 10 |
| ANKRD1 | -4.1 | ankyrin repeat domain 1 (cardiac muscle) |
| FAM111B | -4.0 | family with sequence similarity 111, member B |
| IL7R | -3.9 | interleukin 7 receptor |
| IGHD | -3.8 | immunoglobulin heavy constant delta |
| E2F8 | -3.8 | E2F transcription factor 8 |
| AOC2 | -3.8 | amine oxidase, copper containing 2 (retina-specific) |
| EVI2A | -3.8 | ecotropic viral integration site 2a |
| E2F2 | -3.8 | E2F transcription factor 2 |

| | | |
|---|---|---|
| VAV1 | -3.7 | vav 1 guanine nucleotide exchange factor |
| ADARB1 | -3.7 | adenosine deaminase, RNA-specific, B1 |
| CD274 | -3.7 | CD274 molecule |
| DMC1 | -3.6 | DMC1 dosage suppressor of mck1 homolog, meiosis-specific homologous recombination |
| BIRC3 | -3.5 | baculoviral IAP repeat containing 3 |
| ZNF367 | -3.5 | zinc finger protein 367 |
| KLHL23 | -3.5 | kelch-like family member 23 |
| ARHGAP11A | -3.5 | Rho GTPase activating protein 11A |
| PPARGC1B | -3.5 | peroxisome proliferator-activated receptor gamma, coactivator 1 beta |
| GDF6 | -3.4 | growth differentiation factor 6 |
