# Supplementary material for: Simvastatin Modulates Mesenchymal Stromal Cell Proliferation and Gene Expression
Source: PLoS One. 2015 Apr 13;10(4):e0120137. doi: 10.1371/journal.pone.0120137 (PMC4395223; doi:10.1371/journal.pone.0120137)
Supplement: S3 Table — Average percentage of positive and negative markers for AM-MSC and BM-MSC. (PDF) [file pone.0120137.s003.pdf]

Positive markers			Negative markers	
	Avg (%)			Avg (%)
**CD105**	84,82		**CD14**	0,07
**CD73**	89,45		**CD45**	0,22
**CD 90**	80,02		**CD34**	0,55
			**HLA-DR**	0,49
			**CD19**	0,38
