# Supplementary figures and images for: Simvastatin Modulates Mesenchymal Stromal Cell Proliferation and Gene Expression
Source: PLoS One. 2015 Apr 13;10(4):e0120137. doi: 10.1371/journal.pone.0120137 (PMC4395223; doi:10.1371/journal.pone.0120137)

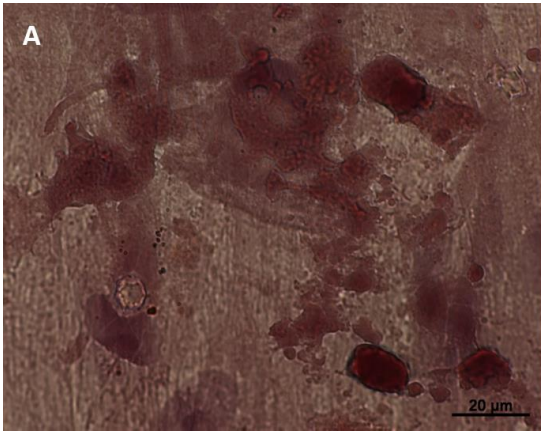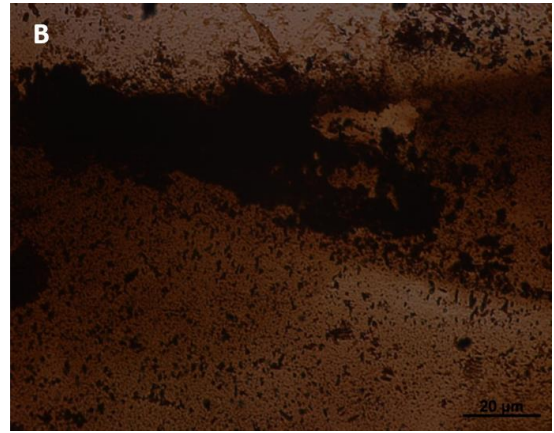

Supplement: S1 Fig — For the differentiations, cells were cultured with specific inductors of differentiation into adipocytes (A) and osteocytes (B). Adipocyte cultures were stained with Sudan II and Scarlet stains and osteocyte cultures were stained with Von Kossa and Harris hematoxilin. (PDF) [file pone.0120137.s004.pdf]

**A**

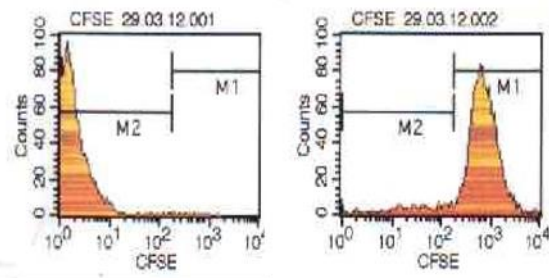

**B**

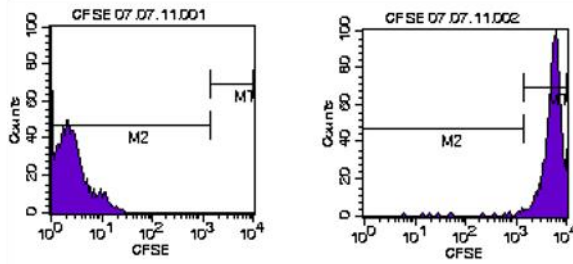

**C**

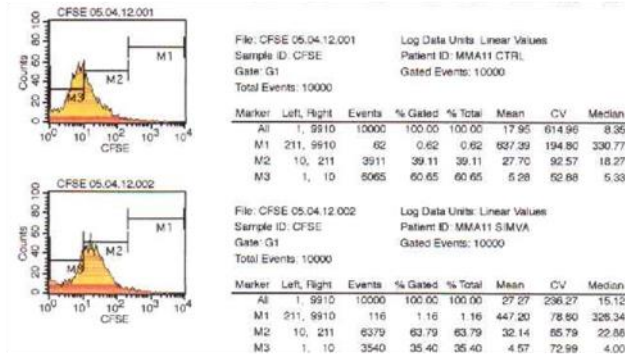

Supplement: S2 Fig — (A) Basal CFSE staining and after co-culture staining of MSC; (B) basal CFSE staining and after co-culture staining of PBMC and (C) representative analysis of MSC CFSE experiment. (PDF) [file pone.0120137.s005.pdf]
